# Exploring the varied manifestations of structural violence in the lives of children on the autism spectrum and their families: a qualitative longitudinal study in Kurdistan, Iran

**DOI:** 10.1186/s12939-023-02078-z

**Published:** 2023-12-18

**Authors:** Ansar Khougar, Paria Baba Ahmadi, Hadi Ranjbar, Mahsa Ahadi, Parisa Ahadi

**Affiliations:** 1 Present Address: Primary Health Care Center, Iran University of Medical Science, Shahryar, Iran; 2 Student Research Committee, School of Medicine, Shirza University of Medical Sciences, Shiraz, Iran; 3 Mental Health Research Center, Iran University of Medical Science, Tehran, Iran; 4 Department of Pharmacy, Ayatollah Amoli Branch, Islamic Azad University, Amol, Iran

**Keywords:** Autism, Child, Families, Structural violence, Iran, Kurdistan

## Abstract

**Background:**

There are many dimensions regarding autism that are closely connected to social structures, policies, and power dynamics, silently impacting the well-being of individuals within the autism spectrum. This research aims to explore these overlooked aspects using a theoretical framework called "structural violence."

**Methods:**

The study was conducted in Kurdistan, Iran, and a qualitative longitudinal approach was chosen. A purposive sampling method was employed to select the participants, with 11 parents taking part. The study data comprised 29 interviews using a topic guide conducted over a span of 2 years. Thematic analysis and a matrix-based approach were utilized for data analysis. To enhance the scientific rigor of this research, four criteria, including Guba and Lincoln’s principles, were implemented to ensure methodological accuracy.

**Results:**

The research findings highlight four primary forms through which structural violence impacts children on the autism spectrum and their families: access to healthcare, geographic disparities, awareness and stigma, and poverty and financial burden. Additionally, the study identified 11 subthemes related to structural violence in the context of autism and families.

**Conclusions:**

We illustrated how structural forces create barriers to accessing adequate healthcare services, exacerbate discrimination based on ethnicity and geography, perpetuate stigma, and contribute to poverty and the inability to meet basic needs. These factors not only worsen health issues but also deepen existing disparities in healthcare access and outcomes for children on the autism spectrum and families. We emphasize the urgent need for systemic changes to address these issues. It is essential to promote public awareness, provide better access to health and support services, and address economic and political factors that contribute to these inequalities.

**Supplementary Information:**

The online version contains supplementary material available at 10.1186/s12939-023-02078-z.

## Background

Autism spectrum disorder (ASD) is recognized as a category of neurodevelopmental disorders [1]. 

Framing autism as a neurodevelopmental disorder can in two ways inadvertently shift focus away from exploring the often overlooked and impactful dimensions associated with autism. Firstly, When defining a disorder and disability, besides physical factors, social factors should also be incorporated [2]. Secondly, the term ‘neurodevelopmental’ may initially guide our attention towards neurological and medical aspects, potentially diverting our focus from exploring the frequently overlooked yet significant dimensions intricately linked to social structures, policies, and power dynamics, which subtly influence the well-being of individuals within the autism spectrum. This research specifically probes these neglected aspects.

Autism affects social interactions and communications. It also causes specialised interests and repetitive behaviors [3]. Autism is a lifelong condition, with its first specific characteristics typically appearing in early childhood [4, 5]. People on the autism specturm experience elevated levels of accompanying medical conditions, increased mortality rates, substantial healthcare costs, and encounter challenges in obtaining well-coordinated medical assistance [6, 7].

Research shows that approximately 1 in 100 children worldwide are on the autism spectrum [8]. In the United States, the prevalence of autism is approximately 1 in 36 children [9]. The differences in prevalence are likely due to a scarcity of diagnostic resources, insufficient follow-up in screening programs, and cultural barriers, such as social stigma [10].

Several factors distinguish autism from other developmental disorders. Having an expected development period followed by specific autistic characteristics, prolonged diagnosis process, High prevalence and challenges in curing co-occurring medical problems, missed prognosis, and undetermined causes of the autism are some instances [11–14].

Considering the mentioned reasons, parents of children on the autism spectrum, who have the most elevated levels of communication with them, endure many challenges [13, 15]. Prior studies indicate that these parents, particularly mothers, experience higher stress levels compared to parents of person without autism and are more susceptible to physical and mental health problems [16–18]. Some studies even suggest that these parents encounter greater stress and psychological issues than parents of children with other developmental disorders [19, 20].

A systematic literature review, encompassing a comprehensive search and screening process, identified 27 relevant studies. These studies revealed that healthcare providers frequently reported limited knowledge, resources, and training regarding autism. Consequently, their ability to effectively address the complex care needs of individuals on the autism spectrum was impeded [21]. Employing a community-based participatory research approach, a study investigated the healthcare barriers faced by individuals on the autism spectrum, individuals with other conditions, and those without autism. Notably, participants on the autism spectrum reported unique and more pronounced healthcare barriers compared to others. These findings highlight the disparity in healthcare access for individuals on the autism spectrum and probe additional barriers specific to their unique needs [22].

The situation in Iran is further complicated by political, economic, and social circumstances [23, 24]. Estimates suggest a 0.92% prevalence of autism in Iran [25]. A study aimed to examine the health of Iranian mothers and fathers with children diagnosed with intellectual disabilities or autism. The results showed that families with children on the autism spectrum exhibited significantly poorer family functioning, with both mothers and fathers reporting heightened levels of stress. Mothers and single parents demonstrated poorer emotional well-being [26].

A systematic review aimed at exploring the lived experiences of Iranian parents with children diagnosed with autism revealed that they encounter several challenges. These challenges include inaccurate diagnostic processes, insufficient support during diagnosis, substandard services, psychological issues, and anxiety about their child’s future. Additionally, the parents’ experiences highlighted family issues, difficulties in engaging in daily activities and stigma [27]. A cross-sectional online survey, assessing stigma and knowledge about autism among the Iranian population, unveiled an insufficient understanding of autism among Iranians [28].

The findings of a study suggested that the top intervention priority for parents of Iranian children on the autism spectrum was the development of social communication skills [29]. In another study, self-care was identified as the primary challenge for children, as perceived by the majority of parents [25]. Iranian parents also seek guidance on managing challenging behaviors in their children, desire quality time with friends and family, and aspire to have routine access to specialists [30]. A study identified several barriers that impede access to care for individuals on the autism spectrum. These barriers include a shortage of qualified professionals, challenges in accessing appropriate services for families, financial burdens, and a lack of formal and informal support systems [31].

This research aims to comprehensively examine the obstacles and issues that Iranian families have mentioned in previous research from various angles, utilizing a theoretical framework called structural violence within a qualitative longitudinal study.

### Theoretical Framework : Structural Violence 

In this section, we focus on a concept that has gained increasing prominence in the twenty-first century, highlighting the profound impact of social, economic, and political factors on individual well-being. Structural violence (SV) encompasses societal frameworks, including economic, health, legal, political, religious, and cultural systems, that hinder individuals, groups, and societies from achieving their fullest potential. This pervasive form of violence can lead to injustices, causing harm and adversely affecting the well-being of individuals [32].

Structural violence (SV) originated from Johan Galtung’s work. Galtung says, ‘When the potential is higher than the actual is by definition avoidable and when it is avoidable, then violence is present’ [32]. It means violence exists when the potential for positive human development falls short of what could be achieved, and this gap is avoidable, meaning it could be eliminated through social, political, or economic changes. For instance, if a society lacks adequate hospitals, preventing people from accessing necessary medical care, this would constitute avoidable deprivation, as it could be addressed by investing in healthcare services.

These preventable barriers imposed on certain groups of people, manifests through established institutions and everyday interactions, leading to unequal access to resources, education, power, and healthcare [33].

SV is particularly prominent in medical anthropology. An anthropologist and physician, Dr. Farmer has worked in this field for decades. SV directly harms the health and access to healthcare for patients and caregivers by overlooking larger structural issues [34]. It has become increasingly evident that without a comprehensive understanding of SV in the context of disease, medical and public health interventions are bound to fall short of achieving their intended goals [35].

Various studies have demonstrated that structural violence can manifest in several ways, including: internal displacement and development; food and politics; water and sanitation; social services [36]; and food insecurity [37]. Additionally, the indefinite detention of individuals with intellectual disabilities under forensic mental health law constitutes a manifestation of SV [38]. SV also affects other vulnerable groups such as immigrants [39] and individuals living with HIV in these communities [40]. Also Gilligan cites the higher mortality rates among lower socioeconomic strata compared to upper strata as a stark example of structural violence [41].

An ethnographic study conducted in Cambodia suggests that structural violence (SV) poses a significant barrier to accessing education in the country. This study involved comprehensive investigations of schools, including on-site visits, engagement with teachers and directors, classroom observations, and conversations with diverse community members. The research also encompassed 12 in-depth interviews with disability rights advocates and consultations with representatives from Cambodia’s Ministry of Education, Youth, and Sports. SV manifests through social exclusion, oppression, and a lack of agency, all of which are deeply embedded within the structures of Cambodian society. The presence of a disability can trigger stigmatization, often leading to discrimination and exclusion [42].

Diana Szanto argues rather than discrimination, it is SV that makes the lives of individuals with disabilities unbearable, and that they are victims of this SV [43]. That is why when defining a disorder and disability, besides physical factors, social factors should also be incorporated.

Critical Disability Theorists argue that disability is not primarily a medical state, but a question of politics and power (powerlessness, power to and power over). In fact, issues related to disability encompass social values, institutions and political priorities, and are not only about impairments [44]. These people are vulnerable to multiple forms of violence in their everyday lives, including structural violence, deprivation, and physical, emotional, and sexual exploitation [45]. Therefore, it is crucial to explore further, the relationship between structural conditions and the experiences of individuals with disabilities [46].

The approach of structural violence has the potential to uncover the underlying dynamics of social practices that operate across various aspects of individuals’ lives, even if they may not seem directly linked to health. The optimal method for doing this is by utilizing real-life examples that closely examine the experiences of individuals directly affected by structural violence [47].

## Methods

We employed LQR because human experiences are not static and consistent, but rather dynamic and diverse. Consequently, individuals’ experiences with a health phenomenon can be captured more holistically through repeated interviews or observations over time [48].

The use of longitudinal data, which tracks practices, perspectives, individual relevancies, and experiences over time, offers substantial advantages in various research fields, including life course, social policy, health, migration, and family research [49]. While longitudinal qualitative research on caregivers and vulnerable people has been conducted previously [50–55], this method holds particular strength in the field of disability by elucidating the impact of environmental or contextual factors on participants’ experiences as they evolve over time [56].

Research has shown that to gain a comprehensive understanding of how politics and policies affect the opportunities and choices available to individuals, it is necessary to observe and track their experiences over an extended period [57]. These methods meticulously examine the subtle nuances and possess the potential to deepen our understanding of intricate social processes and interrelationships that may influence health-related behaviors and outcomes [58, 59].

This point holds true for the issue of SV, as structural and institutional violence is not apparent at first glance. For this reason we can provide a nuanced understanding of the ongoing and changing impact of SV on parents of a child on the autism spectrum. This information can help inform policy and advocacy efforts aimed at addressing the root causes of structural inequities and improving the lives of families affected by autism.

The research was conducted in Kurdistan province. Participants were given the choice of conducting the interviews in their homes, public places, or community centers.

### Context

Kurdistan Province is situated in western Iran, with Sanandaj as its capital city. The majority of the province’s inhabitants belong to the Kurdish ethnic group, who speak the Kurdish language and predominantly adhere to the Sunni branch of Islam. This distinct religious and ethnic heritage sets them apart as a minority group within Iran. The province has an approximate population of 1.6 million people according to official population statistics. A research study conducted in Kurdistan reported a 0.1% prevalence rate for autism [60].

### Sampling and participants

In this study, purposive sampling was employed to select the participants. Researchers reached out to families through autism associations, extending an invitation to participate in interviews. These associations, predominantly non-governmental organizations (NGOs) operating as charities, offer limited educational support and self-care skills training. Sixteen parents were invited to participate in the study, however, five declined. Ultimately, 11 parents (four fathers and seven mothers) of children on the autism spectrum between the ages of 2 and 6 at the time of the first interview took part in the research. Among the children, four were female and seven were male. All participants were Kurds and residents in Kurdistan. The parents’ ages ranged from 31 to 55, with eight being married and three being single. Table 1 shows the characteristics of the participants.

**Table 1:** The characteristics of the participants

**Number**	**Participants**	**Age of parents**	**Parent Marital Status**	**Sex of child**	**Age at the first Interview**	**Age at diagnosis**
1	Zanko’s father	55	Married	Female	2.5	2
2	Zhina’s mother	49	Married	Female	4	3
3	Mohammad’s father	39	Married	Male	3	2.5
4	Hemen’s mother	42	Married	Male	3.5	2.5
5	Sirvan’s mother	29	Single	Male	6	5
6	Aso’s mother	45	Married	Male	5	4
7	Hana’s father	34	Single	Female	3	2
8	Kani’s mother	35	Married	Female	2	1.5
9	Armin’s mother	37	Single	Male	2.5	2
10	Samyar’s mother	32	Married	Male	4	3
11	Zhian’s father	31	Married	Male	5.5	4.5

The following criteria were used to enter this research:

1. Parents of a child diagnosed with autism who are responsible for taking care of their child.
2. Kurd and resident in Kurdistan
3. The child is between the ages of 2 and 6

### Procedure and data collection

The study’s data consisted of 29 interviews conducted with 11 parents over a period of 2 years. The research tool utilized for this purpose was a semi-structured in-depth interview. The interviews were conducted at three different time points: 6, 18, and 30 months after the diagnosis. Initially, the first interview was supposed to be conducted after the diagnosis. However, after a decision with the authors and considering the complexity of the diagnostic process, it was decided to conduct the first interview 6 months after the diagnosis. The research and interview process spanned from August 2018 to February 2021. The interval between data collection points provides the research team with the chance to review and adjust interview guides. This enabled us the design of subsequent interviews to build upon, rather than duplicate, the data previously collected [61].

To ensure the highest quality data collection, a community mental health researcher with expertise in interviewing vulnerable populations oversaw and guided the interviews. Additionally, the interviewers selected for the study were experienced nurses with a background in working with families facing similar circumstances.

A separate topic guide was designed for each interview round (See Additional File 1). The first guide was based on a thorough review of recent literature and guidance from Strategies for Qualitative Interviews. When designing subsequent guides, the analysis of previous interviews was also incorporated to further refine the focus and direction of the interviews [61].

Subsequent interviews commenced with the interviewer providing a brief summary of the key takeaways from the previous session, highlighting the emerging themes that had been identified. Participants were then invited to reflect on this summary of experiences before proceeding with the structured interview questions. This approach significantly enhanced the focus and coherence of the data collected, thereby streamlining the analysis process [62].

### Analysis

Janet Holland emphasizes the importance of time, process, and change as essential components in qualitative longitudinal research [63]. In this study, we took care to ensure that the analysis effectively incorporates and captures these elements, acknowledging their critical significance.

The initial step in analyzing data in LQR often involves a cross-sectional analysis [62]. We employed thematic analysis, a technique that identifies and analyzes patterns and themes while meticulously organizing and describing information [64]. We employed MAXQDA for this, a software designed to facilitate various qualitative methods. Its functionalities include tasks such as transcription, coding frame construction, concept map creation, and group comparisons [65].

In order to capture the comprehensive and profound nature of participants’ narratives, all interviews were meticulously recorded and transcribed word for word. These transcripts were then carefully cross-checked against the audio recordings to ensure accuracy and fidelity.

Synchronic analysis was conducted throughout each wave of data collection [66]. The data analysis process commenced from the very first interview. Firstly, we familiarized themselves with the data by reading and re-reading it to gain a comprehensive understanding of its content. After team discussions on data quality the transcripts were then organized and managed. Then, coding the data, identifying patterns, themes, and categories that emerged from the data itself was started. This involved systematically labeling and categorizing segments of data based on their relevance to specific themes or concepts. After coding, the Researchers organized the codes into potential themes, grouping together related codes that captured similar ideas or patterns within the data. The themes were reviewed, refined, and named to accurately represent the content and meaning of the data [67].

Then we used a matrix-based approach to develop a longitudinal description of the themes and analyze changes [68]. We created an overall matrix for all participants and individual matrices for each participant. The analysis involved comparing and contrasting themes, exploring variations or subthemes within larger themes, identifying any relationships or connections between themes, and tracking changes [69] (see Tables 2 and 3). The matrices displayed subthemes in rows and each wave in columns. Each matrix presents the number of codes applied to each subtheme By analyzing the rows of the table, we can track the changes of themes across time.

**Table 2:** Matrix for all Participant in 3 waves of the interviews

**Theme**	**Sub-Theme**	**1st wave**	**2nd wave**	**3nd wave**
Access to Healthcare	Diagnostic challenges	16 code	6 code	4 code
	Specialized Medical Services and Facilities	14 code	18 code	22 code
	Healthcare Coverage	12 code	16 code	16 code
Geographic Disparities	Health Policy and system support	12 code	14 code	18 code
	Public Schools and ASD Care Centers	8 code	12 code	18 code
	Migration	0 code	6 code	10 code
Awareness and Stigma	Public Awareness and Stigma	6 code	10 code	18 code
	Isolation and Social Exclusion	6 code	14 code	22 code
	compound impact on women	8 code	12 code	12 code
Poverty and Financial Burden	Basic needs	10 code	18 code	22 code
	Child’s progress	6 code	8 code	12 code

**Table 3:** Matrix Participant 1 in 3 waves of the interviews

**Theme**	**Sub-Theme**	**1st wave**	**2nd wave**	**3nd wave**
Access to Healthcare	Diagnostic challenges	4 code	1 code	2 code
	Specialized Medical Services and Facilities	1 code	1 code	2 code
	Healthcare Coverage	1 code	1 code	2 code
Geographic Disparities	Health Policy and system support	2 code	2 code	3 code
	Public Schools and ASD Care Centers	2 code	1 code	2 code
	Migration	0 code	0 code	0 code
Awareness and Stigma	Public Awareness and Stigma	2 code	1 code	2 code
	Isolation and Social Exclusion	0 code	1 code	3 code
	compound impact on women	0 code	0 code	0 code
Poverty and Financial Burden	Basic needs	1 code	2 code	2 code
	Child’s progress	0 code	0 code	0 code

Four criteria, including Guba and Lincoln, were used to increase scientific accuracy in this research. Guba and Lincoln proposed reliability or trustworthiness criteria for evaluating the results of qualitative research to understand the extent to which we can rely on the results of a qualitative study [70].

## Results

**Fig. 1:**
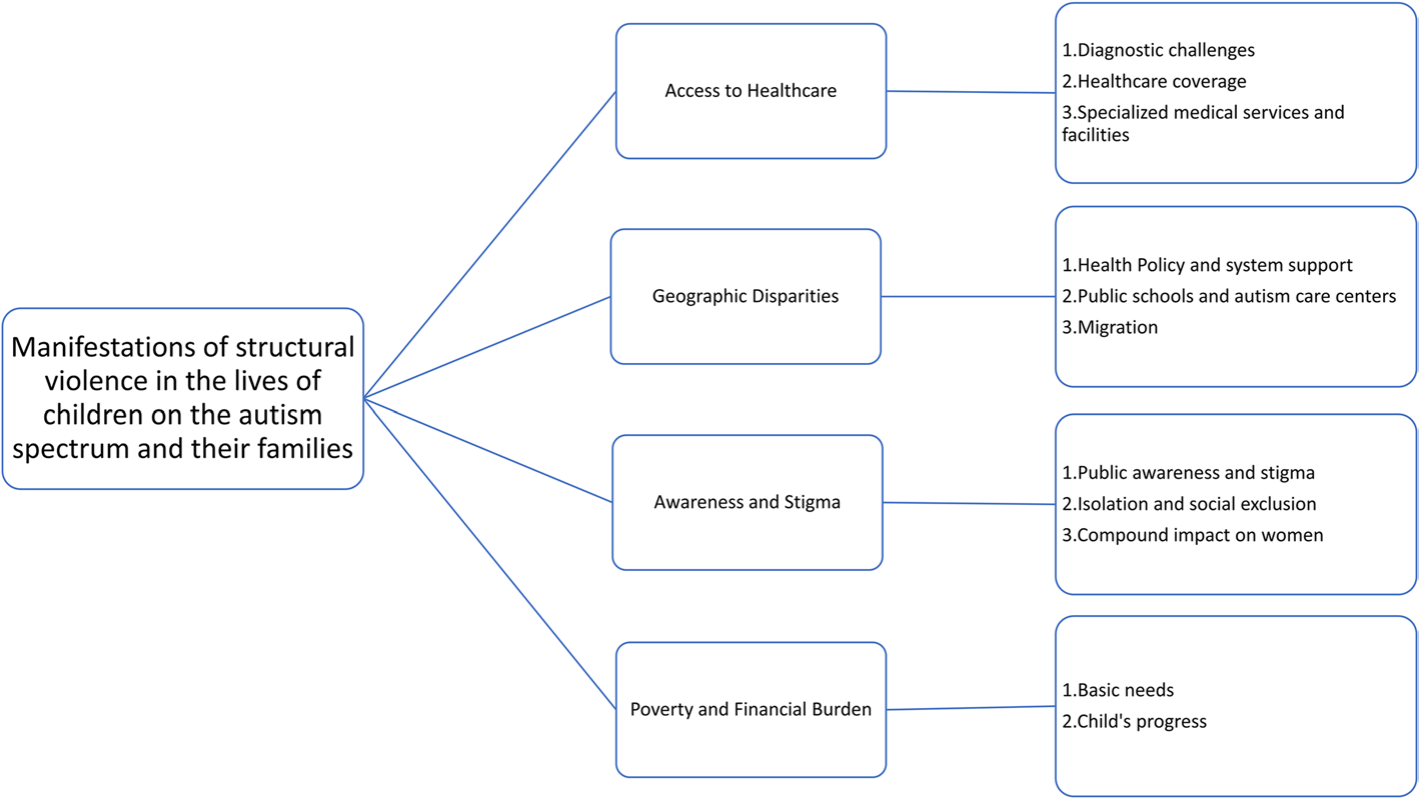
Theme and subthemes

As shown in Fig. 1, the findings reveal that structural violence manifests in four forms concerning children on the autism spectrum and their families:

1. Access to Healthcare
2. Geographic Disparities
3. Awareness and Stigma
4. Poverty and Financial Burden

These four themes provide us with valuable insights into the health and well-being of children on the autism spectrum and their families. Through a descriptive lens of SV, we can better understand the challenges they face. Moreover, exploring the intersectionality between ethnicity and disabilities and tracking changes over time helps us comprehend how these factors shape their experiences. In the following section, each of these themes and their sub-themes will be provided along with quotes from the participants.

To make the data more meaningful, Table 4 presents the percentages and frequencies for the extracted themes and subthemes.

**Table 4:** The percentages and frequencies of themes and subthemes

**Theme**	**Sub-Theme**	***n***	**%**	**Participant number**
Access to Healthcare	Diagnostic challenges	11	100	1,2,3,4,5,6,7,8,9,10,11
	Specialized Medical Services and Facilities	11	100	1,2,3,4,5,6,7,8,9,10,11
	Healthcare Coverage	11	100	1,2,3,4,5,6,7,8,9,10,11
Geographic Disparities	Health Policy and system support	11	100	1,2,3,4,5,6,7,8,9,10,11
	Public Schools and autism Care Centers	11	100	1,2,3,4,5,6,7,8,9,10,11
	Migration	3	27.2	2,10,11
Awareness and Stigma	Public Awareness and Stigma	11	100	1,2,3,4,5,6,7,8,9,10,11
	Isolation and Social Exclusion	11	100	1,2,3,4,5,6,7,8,9,10,11
	compound impact on women	7	63.6	2,4,5,6,8,9,10
Poverty and Financial Burden	Basic needs	9	81.8	1,2,3,4,5,6,9,10,11
	Child’s progress	8	72.7	2,3,4,5,6,9,10,11

### Theme 1: access to healthcare

#### Diagnostic challenges

Getting an autism diagnosis is tough and can be a lengthy journey for families. Parents often struggle due to limited knowledge about autism, confusion about when the diagnosis happens, different diagnosis and delay in diagnosis. Sirvan’s mother recounts her experience, saying, “When I called him, he didn’t react to the sound, so I suspected he might be deaf and took him to an audiologist. However, there was no problem found, and they didn’t provide any further guidance.” (1st round).

Armin’s mother shares, “I noticed that my third child, Armin, was different from my other children since he was 9 months old. However, I had no information about autism, and none of the doctors diagnosed him.” (1st round). Late diagnosis is often linked to high costs and extensive tests, which add to the challenges faced by families. Zhian’s father adds, “I spent a significant portion of my salary until we realized the issue.” (1st round).

#### Healthcare coverage

Another challenge arises when individuals seek diagnosis and support: limited or nonexistent health insurance coverage for certain medical conditions or specialized support or services can hinder their access to specialized medical services and facilities. Sara explains, “Even with insurance, many medications are not covered. The process of obtaining covered medications is complicated, and people often struggle to navigate through it. I remember one time when I went to get medication, I was sent to different rooms and offices multiple times. Eventually, I became overwhelmed and ended up sitting in the middle of a hallway, crying.” (1st round).

Mohammad’s father says, “Despite the constant interviews claiming that autism is covered by insurance, we have not seen anything of the sort. We are unemployed and do not have any insurance to cover our expenses.” (2nd round).

#### Specialized medical services and facilities

One major obstacle is the limited availability of specialized medical services, making it hard for parents to find healthcare providers with expertise in diagnosing and supporting autism. Zhina’s mother emphasizes that speech therapy alone is not sufficient; they also need neurologists, psychiatrists, and occupational therapists with specialized knowledge and skills. (2nd round). Moreover, the absence of dedicated hospitals for autism services worsens the challenge of accessing appropriate care. Specialized facilities are scarce and difficult to find, and many services are not available locally.

Aso’s mother says: “Once, Aso was experiencing an ache in his mouth, and I realized it might be related to his teeth. I had never thought about these kinds of problems before, so I took him to a dentistry clinic. However, they were unable to provide any treatment due to Aso’s condition. The lack of specialized dental services in our area forced us to travel to other provinces for dental treatments.” (3rd round).

### Theme 2: geographic disparities

#### Health policy and system support

Geographic disparities in Kurdistan create significant challenges for children on the autism spectrum and their families. Inadequate policies and financial resource limitations hinder access to crucial areas such as early intervention services, education, employment opportunities, healthcare access, and social inclusion.

Samyar’s mother said, “My child has both autism and epilepsy, and I give him numerous medications daily. Aside from the financial aspect, our main problem lies in the unavailability of medications. We have to travel to the provincial center or even other provinces to obtain the medications.”

Kani’s mother shared, “Our city only has one hospital, and it does not provide any specialized services for autism. We are forced to travel to other provinces for an MRI or CT scan, and even then, there is no proper transportation system.” (1st round).

Mohammad’s father explicitly stated, “It is not just about geography. From what I can remember, the Kurdish population has consistently faced more disadvantages.” (3rd round).

Additionally, the lack of funding allocated to programs, services, and research dedicated to healthcare was prominent in the interviews. Zhian ‘s father said, “Every time we went to the welfare or health network for help, they said we could not.” (3rd round) According to the parents, limited funding has led to protracted waiting lists for assessments and interventions, understaffing in educational settings, a scarcity of specialized healthcare providers, and restricted access to social resources and support networks.

#### Public schools and autism care centers

The increase in the number of codes for the sub-theme of “Public Schools and autism Care Centers” from 8 in the first wave to 18 in the third wave highlights the growing concern among families regarding the adequacy of educational resources for children on the autism spectrum. The dearth of public schools and autism care centers has several consequences that impact individuals on the autism spectrum and their families. It can curtail educational opportunities, delayed access to necessary interventions and therapies, and increased burden on families. In the absence of readily accessible and suitable public options, families often turn to costly private alternatives, but due to the prohibitive expense of these private services, many families are ultimately unable to obtain the necessary support.

Samyar’s mother mentioned that Samyar does not attend school and receives education at home. (3rd round).

These challenges also affect social interactions. Samyar’s mother desires to go out or attend parties with her spouse, but finding someone to care for their son poses difficulties. Establishing government-operated centers would greatly assist families in such situations.

Mohammad’s father mentioned the significant distance to the nearest rehabilitation center, leading to higher expenses. In the 3rd round of interview, it was revealed that education for the child has been halted due to these limitations. In fact transportation poses further challenges for these families. Zanko’s father accurately describes the situation, saying, “Commuting is a major challenge for us. Public transportation is inadequate, and our child is greatly affected by this inconvenience. Even traveling to other provinces for medical treatment presents problems. Unfortunately, we lack train services, and flights are limited to once or twice a week. Affording them may be beyond our means.” (1st round).

#### Migration

Autism can have a profound impact on families, leading to significant changes such as relocation or changes in employment. In some cases, the father, who is typically responsible for the family’s income, may need to seek employment in other cities or even in Kurdistan, Iraq, and be separated from the family for a few months. Samyar’s mother in (2nd round) shares that her husband has traveled to Tehran in search of work and is currently employed there as a laborer. Similarly, Zhina’s mother has faced a similar situation where her husband, years after diagnosis, had to relocate to one of the cities in Kurdistan, Iraq, in search of employment. (3rd round).

However, the story does not end there, and sometimes the only viable solution is for the entire family to migrate together in pursuit of a better life. Due to the lack of adequate medical and educational facilities, some families in the Kurdistan province have been compelled to migrate to the central part of the province and Tehran. In the 3rd wave of interview, Zhian’s father expresses that they can no longer remain in their current location, and it is highly likely that they will move to Tehran and find a home in one of its cities within the next few months.

### Theme 3: awareness and stigma

#### Public awareness and stigma

Autism awareness remains low among families and society at large. Authorities’ inadequate efforts in identifying autism lead to a plethora of challenges for families. Limited public awareness and stigma, as well as a lack of training and knowledge about autism among healthcare professionals, contribute to these challenges. The feeling of isolation and social exclusion further compounds the difficulties faced by families.

Hajir’s mother highlights the significant lack of awareness, stating, “Even after explaining, people still have a limited understanding of autism.” (2nd round).

The limited awareness within families can pose challenges in finding appropriate healthcare professionals, therapists, and specialized educational resources for their child. Armin’s mother shares her initial confusion, stating, “I didn’t know where to seek help, which school to choose, which department in the hospital to approach, or which specialist to consult.” (1st tound).

The lack of awareness is not confined to the general public or families alone; even medical staff, professionals, and individuals working within the healthcare system may act out of ignorance, leading to the perpetuation of stigma.

Zanko’s father shares his experience of frequently engaging in conflicts with the hospital guards. He expresses his frustration, emphasizing, “ My child is unable to stay in the waiting room and often starts to cry and scream, but the personnel fail to understand the urgency of the situation.” (2nd round).

Zhian’s father recounts an unfortunate incident when enrolling Zhian in school. He remembers, “Despite my son not feeling well during the screening, they instructed us to perform certain tasks which Zhian could not do. Subsequently, an official suggested, Take him to Disability Care Center and reside there.” (2nd round).

#### Isolation and social exclusion

Regarding this situation, Hemen’s mother emphasizes that children on the autism spectrum face challenges in crowded places, making even simple tasks like going to the hospital exceedingly difficult. She believes their only option is to withdraw or retreat. This issue also extends to other activities, such as using public transportation, where families dealing with autism struggle. (2nd round).

Aso’s mother shares her experience, stating that her son actively avoids crowded places and gatherings, which means they cannot attend gatherings or parties. (2nd round).

Armin’s mother explains their situation, mentioning the difficulties they face in going to new places due to her son’s restlessness in unfamiliar environments. His response of engaging in repetitive movements adds to the challenges they encounter. (3rd round).

Hana’s father mentioned that we used to always go on picnics with other relatives during holidays, but now it has become very difficult for us to do so. (2nd round).

#### Compound impact on women

The demands of caring for a child on the autism spectrum can be especially challenging for women. Women often bear a disproportionate responsibility for caregiving, leading to increased emotional and physical stress, financial strain, and social isolation. Additionally, societal expectations and gender roles can present additional obstacles, making it harder for women to balance caregiving with work and personal responsibilities.

Aso’s mother has noticed that she is unfairly blamed and held solely responsible for things, causing her distress. (2nd round) Similarly, Armin’s mother says: I experience blame from both strangers and those close to me, despite the extensive duties and work I undertake. (3rd round).

### Theme 4: poverty and financial burden

#### Basic needs

Fulfilling basic needs presents significant challenges. Over time, the family’s dietary habits and recreational activities undergo noticeable changes. These changes not only impact parents but also other siblings, whose recreational opportunities are curtailed. Some parents mentioned that their food basket has changed, and they can no longer afford to spend on their own meals. Additionally, their children have different food preferences.

Aso’s mother stated, “In the past 2 years, I clearly see that the consumption of meat and protein in our home has decreased.” (3rd round).

Housing instability is another problem. Having a separate and stable home is crucial because children may produce loud noises that can cause issues. However, due to the families’ economic situation, they may not even have a separate apartment, let alone a villa.

Mohammad’s father shared, “Last year, I had to sell the house to cover medical expenses, and now I’m renting.” (3rd round).

#### Child’s progress

The financial burden associated with autism is so significant that it hinders families from accessing necessary medical services. Aso’s mother expressed her concerns, saying, “When the autism was diagnosed, we started the support or services immediately. Initially, I took him to the specialist 3 days a week, but now we can only manage 1 day a week due to the high costs involved.” (3rd round).

Hemen’s mother also shared her perspective, saying, “Even when we had an Allistic child, our financial situation was not favorable enough to afford the expenses. Now, with the added burden of autism-related costs, it has become nearly impossible for us.” (2nd round).

Armin’s mother said, “My husband works as a kolber.^1^ The income earned as a kolber is not stable or sufficient to meet the high expenses associated with our child’s condition.” (1st round).

## Discussion

Raising a child on the autism spectrum presents challenges and complexities. In this research, we examined whether children on the autism spectrum and families are exposed to structural violence or not and how they are impacted. Excluding the “Diagnostic Challenges” subtheme, the number of codes for all other subthemes increased from Wave 1 to Wave 3, indicating a rise in the challenges. In the context of person on the autism spectrum and their families, SV manifests in four main forms: Access to Healthcare, Geographic Disparities, Awareness and Stigma and Poverty and Financial Burden. Limited access to healthcare identified as a form of SV in many studies [71, 72]. The diagnostic journey for autism is often a complex and protracted process. Parents expressed frustration with the lack of comprehensive knowledge about autism, which often led to confusion upon receiving the diagnosis. Early Specific autistic experiences and characteristics of autism are frequently overlooked or misinterpreted, delaying the diagnosis and appropriate intervention. Late diagnosis can be attributed to the high costs associated with the diagnostic process, further exacerbating the challenges faced by families. Expensive and extensive tests contribute to financial burdens and can limit access to timely intervention, potentially affecting the child’s developmental outcomes. These findings have been reported in other studies [73, 74]. A study conducted on 17 black individuals with HIV, who were mainly recognized as recent immigrants to Canada, revealed that their inability to access healthcare services could be considered a form of SV. The inadequate knowledge of these individuals about the organization of the Canadian healthcare system poses a challenge to their ability to utilize HIV-related healthcare services [75].

The issue of healthcare coverage presents another significant barrier for families seeking diagnosis and support or services for children which can impede individuals’ access to essential medical services and facilities. Participants in the study highlighted the complex bureaucracy involved in obtaining covered medicines, often leading to unsuccessful attempts. Furthermore, some families, like Mohammad’s, face the additional obstacle of being uninsured, which further limits their access to necessary healthcare services.

The majority of rehabilitation interventions offered at private autism and disability rehabilitation centers are not covered by insurance [76]. The Welfare Organization in Iran provides a small subsidy to its related autism rehabilitation centers, which is the only financial support available [77]. In fact, the insurance services program has allocated only 3% of the total credits of the Welfare Organization to itself [78]. Furthermore, one of the significant changes in the 2023 budget bill in the rehabilitation sector was the removal of the independent budget item “Support for the Rights of Disabled Persons” [78]. Given that institutions are responsible for allocating and removing funds, this issue can be viewed as a manifestation of SV. According to other studies Insurance issues can be identified as a structural violence [75].

Participants emphasized the necessity of involvement from healthcare professionals with expertise in diagnosing and supporting autism. This includes professionals such as neurologists, psychiatrists, speech therapists, and occupational therapists. However, finding healthcare providers with the necessary specialization can be challenging, exacerbating the difficulties faced by families. Furthermore, the absence of dedicated hospitals or services centers specifically for autism further compounds the issue. Families often have to travel long distances or even to different provinces to access specialized care. This not only adds to the financial burden but also creates logistical challenges and limits the availability of services within the local area. Aso’s mother highlighted the lack of dental services, which forces them to seek dental treatment in other provinces, emphasizing the scope of the problem. This highlights a deep-seated problem experienced by other families in various ways, highlighting the issue of Geographic Disparities.

The geographical location plays a crucial role in comprehending the discrepancies in healthcare and social service access, particularly for populations with low socioeconomic status [79]. It has been shown that the majority of underdeveloped and marginalized cities in Iran are located in border and peripheral regions [80]. The findings of this study highlighted the geographic disparities in providing support and care for individuals on the autism spectrum in the cities of Kurdistan province. In fact, these regions even struggle with providing healthcare and support services for other illnesses, let alone for children on the autism spectrum [81]. Also a study revealed the presence of socioeconomic disparities in both the quality of and access to services for children on the autism spectrum in the North-West region of Iran [82]. It is worth mentioning that some participants in the study highlighted that health disparities are not solely attributed to geography but are also influenced by ethnicity.  A study showed white children on the autism spectrum had more access to a larger variety of services and were more satisfied with their healthcare compared to minority groups [83].

These disparities are considered unjust, and addressing them requires the implementation of preventive measures to mitigate their impact [84]. In general, minority ethnic groups tend to have poorer health outcomes than the overall population [85].

There is an urgent need to reassess health policy and system support in Kurdistan. A research study, which involved healthcare service users, academic graduates, and healthcare providers, highlighted significant concerns regarding disparities in accessing and utilizing healthcare services in Kurdistan [81] another study conducted in Kurdistan identified a potential strategy for promoting the healthcare transformation plan, suggesting a need for changes in policy-making and governmental perspectives [86]. One of the challenges associated with policymaking is the issue of budget allocation. Insufficient budget leads to long waiting lists for assessments and interventions, understaffed educational settings, a shortage of specialized healthcare providers, and limited access to social resources and support networks. The budget allocated to Kurdistan province in 2022 was less than 2 million Rials per person, which is considered low compared to other provinces [87]. This budget shortfall is particularly notable in Kurdistan University of Medical Sciences, which is responsible for healthcare and treatment. A study was conducted to analyze the budget of this University from 1997 to 2018. The results showed that the approved budget for the university in the areas of healthcare, education, and infrastructure did not follow a logical trend and did not correspond to the country’s budget growth and inflation rate [88].

As children on the autism spectrum progress through their developmental stages, their educational needs evolve, necessitating specialized support and accessible care centers. While there are several schools and centers for children on the autism spectrum in the capital and some other cities of Iran, there is a lack of schools in Kurdistan. This is significant because previous studies have shown that access to education can be used as an intervention to reduce the consequences of SV [35]. Another challenge is associated with transportation and related infrastructure. Research has emphasized the inadequate development of communication infrastructure, including railways, airports, and highways, which creates barriers to accessing underserved areas, such as Kurdistan province [89]. These problems significantly impact parents’ ability to access essential services.

These circumstances impose the necessity for parents to seek services and employment opportunities in other cities or provinces. The number of codes for the sub-theme of “Migration” has increased from 0 in the first wave to 10 in the third wave. This suggests that more and more families are migrating in search of better services and support, as confirmed by other studies [90]. It highlights the need for comprehensive and accessible healthcare and educational infrastructure in regions like Kurdistan to prevent families from having to make such difficult choices or undergo prolonged separation. Efforts should be directed towards developing local resources and improving the availability of specialized services to alleviate the burden on families and provide a better support system for children on the autism spectrum and their parents.

The theme of awareness and stigma encompasses various aspects, including public awareness and stigma, healthcare professionals and personnel, isolation and social exclusion as well as pressure on women.

The study reveals a low level of awareness about autism among both the public and families. Authorities and institutions make minimal efforts to identify and understand Autism, leading to numerous challenges for families. Furthermore, the lack of awareness extends beyond the public and families alone. Even medical staff, professionals, and individuals within the healthcare system may contribute to stigma and discrimination due to their lack of understanding. Distressing accounts from parents highlight unfortunate incidents where healthcare professionals demonstrate a lack of empathy and knowledge regarding autism. Findings from a study in Iran revealed persistent misconceptions about Autism related to developmental, cognitive, and emotional aspects among both healthcare workers and pediatricians [91]. These encounters intensify the difficulties faced by families, impeding their access to essential healthcare services and support.

In a cross-sectional study, a sample of 980 Iranian adult Kurds residing in Kurdistan province was examined. The findings revealed that around 50.4% of the population had limited health literacy, while 34.0% exhibited moderate health literacy, and 15.6% demonstrated high health literacy. Moreover, the majority of participants, specifically 60.2%, received poor scores in terms of health information access, and 74.1% obtained low scores in the individual empowerment aspect [92]. Here potential is higher than actual and it is avoidable [32]. the government can increase health literacy and combat stigma through public media, which can help to reduce stigma, but it is still present. For this reason, we can classify this issue as a SV.

As a result, parents of a child with autism experience stigmatization and discrimination, as negative stereotypes and beliefs about autism persist. This negatively impacts the health, well-being, and overall quality of life for both parents and their children. A research conducted in South Africa has acknowledged stigma as a form of SV, particularly in the context of healthcare. The study highlights that stigma from healthcare providers acts as a barrier to accessing necessary healthcare services [93] stigma can result in individuals with disabilities or their families choosing not to disclose their condition, thereby hindering their participation in early treatment, training courses, or educational programs that are essential for their overall well-being [94].

Low awareness and stigma surrounding autism leads to the withdrawal of families from society and social activities, as highlighted in previous studies [95, 96]. This avoidance behavior hampers their ability to engage in typical social experiences, leading to feelings of exclusion and isolation. Moreover, the lack of adequate social support systems further amplifies their vulnerability [97]. Additionally, the challenges faced by individuals with autism in adapting to unfamiliar environments further restrict their opportunities for social engagement, exacerbating their sense of exclusion and isolation. Addressing these challenges necessitates the creation of inclusive environments and the implementation of support mechanisms that cater to the unique needs of individuals with autism [98].

Women may face additional challenges due to societal expectations and gender roles, making it harder to balance the demands of caregiving with work and personal responsibilities. In their daily lives, women in Kurdistan often face unequal and unfair obligations that are accepted as natural, resulting in a state of vulnerability to structural inequalities and violence. The lower position of women in the cultural hierarchy makes them particularly susceptible to gender-based inequalities and violence [99].

Structural inequalities, due to the intersection of gender with poor health, inadequate education, and insufficient care and other condition have a compounded impact on women [100]. The societal structures that exist systematically contribute to the widespread prevalence of sexism in Iran, known as patriarchy. This system, in turn, gives rise to patterns of violence, discrimination, and marginalization against women. The shaping of gendered violence through structural violence leaves women in vulnerable positions [101].

Cultural and traditional customs can contribute to the perpetuation of SV, whether in religious, class-based, or caste-based contexts, or through other forms of prejudice. Cultural perpetuation of gender stereotypes that restrict women’s educational and economic opportunities exemplifies avoidable deprivation, addressable through social and educational reforms.

The final theme is Poverty and Financial Burden. there is a well-established link between disability, poverty, violence, and health [36, 45]. Poverty is extreme, structural, systemic and sustained economic deprivation, which in the first instance typically produces powerlessness. Paul Farmer highlights that when power is distributed through systems in a way that limits individual human agency to the point where it becomes impossible to meet basic human needs, this can be seen as a violation of human rights [102].

Poverty and financial burden have a profound impact on families raising children on the autism spectrum, affecting their basic needs, nutrition, housing stability, and access to necessary medical services. Some parents mentioned the unstable and insufficient income earned through occupations such as kolber, further exacerbating their financial difficulties. This highlights the precarious financial situation faced by families raising children on the autism spectrum and the strain it places on their ability to provide proper care and support and is a form of SV.

Johan Galtung outlines the various forms and mechanisms of SV, which include exploitation, penetration, segmentation, marginalization, and fragmentation [103]. According to Galtung, exploitation is rooted in unjust economic and social relationships, and often occurs within complex systems that involve lengthy and convoluted chains of legislation and cycles. In such situations, underprivileged individuals or those excluded from development suffer from severe disadvantages and forced to live in a state of permanent poverty, often leading to malnutrition and illness. Penetration restricts the underprivileged’s perception of reality, obscuring the true power dynamic between the strong and the weak. In addition, the segmentation results from two processes: marginalization and fragmentation. Both marginalization and fragmentation push the underprivileged to the margins of society, where they are regarded as insignificant, divided, and kept apart from one another.

This applies to families. Some of them find themselves in a state of poverty, struggling to meet their basic needs, including access to proper nutrition. They are forced to migrate, becoming separated from others and turning into marginalized individuals in the destination city, who hold little importance for the system. Even those who remain will continue to be marginalized, just as before. Sadly, these situations ultimately impact children. Specifically, the child’s growth and progress can be hindered either because their needs are not being met or due to financial reasons and lack of access to education. Galton says if insight and/or resources are monopolized by a group or class or are used for other purposes, then the actual level falls below the potential level, and violence is present in the system. We observed this issue in all the findings of this study.

## Conclusions

We illustrated how structural forces create barriers to accessing adequate healthcare services, thereby exacerbating health issues and deepening existing disparities. Overall, the relationship between raising a child on the autism spectrum and SV is complex and multifaceted. While raising these children can be challenging on its own, the additional burden of SV can exacerbate these challenges and make it more difficult for parents to access the resources and support they need. The government and responsible organizations have not had the appropriate focus and plans for these children and their families, and the majority of support and assistance is provided by charitable institutions, autism associations, and community organizations.

Institutions such as education, employment, housing, and healthcare are all areas of l and civic life that have been unable to provide adequate support. It is noteworthy that these areas are interconnected, and improving one of them leads to the improvement of others.

Although Iran boasts a robust primary healthcare system, with health houses extending even to remote villages, the services required by individuals on the autism spectrum, such as rehabilitation services, are mostly delivered at higher levels of care. Consequently, this system may not play a significant role in addressing the needs of autistic individuals in this context.

In conclusion, the study highlights the challenges faced by families with a child on the autism spectrum in Iran and emphasizes the urgent need for systemic changes to address these issues. It is essential to promote public awareness, provide better access to health and support services, and address economic and political factors that contribute to these inequalities. By doing so, we can strive to improve the lives of families with a child on the autism spectrum and create a more equitable society.

## Strengths, limitations and suggesion

This research distinguishes itself in three key aspects. Firstly, it employs a longitudinal qualitative method that has not been previously utilized in autism research conducted in Iran. Secondly, it focuses on minority groups and individuals residing in locations outside of Iran’s capital city, where access to various facilities and services is comparatively limited compared to the capital. Thirdly, it incorporates a theoretical framework that was previously unavailable in Iranian studies and delves into structural issues rather than solely focusing on individual-level concerns.

There are certain limitations to our study. Autism is a highly heterogeneous conition, and the study may not have thoroughly addressed the diverse functional and behavioral characteristics of children on the autism spectrum, affecting parents. Furthermore, a shortage of trained evaluators and the absence of standardized diagnostic methods, impacting the diagnostic process, also influence access to children on the autism spectrum. For a more comprehensive understanding, future research should include the viewpoints of other family members, beyond the primary caregiver. Additionally, conducting a more extensive and diverse study is crucial to validate or refine these initial findings. Findings may be limited to a specific region or cultural context, and generalizing results to broader populations should be done cautiously.

Enhancing the scope and diversity of research by including children on the autism spectrum from diverse socioeconomic backgrounds, ethnicities, and geographic locations, Developing context-specific diagnostic tools and implementing rigorous evaluator training programs, Gathering insights from multiple family members beyond the primary caregiver, Employing a combination of qualitative and quantitative methodologies for a comprehensive understanding, and Analyzing resource allocation to identify disparities and inform equitable distribution could be considered for future research.

Also Further research is warranted to elucidate the role of the primary healthcare system in the early identification, screening, and intervention strategies for autism.

### Supplementary Information

**Additional file 1.**

## Data Availability

All the audio files and transcripts of the interviews are currently in possession of the authors and can be provided upon request by the magazine. However, to protect the participants’ information and ensure preservation, the original files have not been made available to the general public.

## Abbreviations

SV: Structural Violence
LQR: Longitudinal Qualitative Research

## Footnotes

1 kolbar or cross-border labor is a worker who is employed to carry goods on his/her back across the borders of Iran and Iraq
