# Supplementary material for: Exploring the varied manifestations of structural violence in the lives of children on the autism spectrum and their families: a qualitative longitudinal study in Kurdistan, Iran
Source: Int J Equity Health. 2023 Dec 18;22:263. doi: 10.1186/s12939-023-02078-z (PMC10729435; doi:10.1186/s12939-023-02078-z)
Supplement: Supplementary file 1 — Additional file 1. [file 12939_2023_2078_MOESM1_ESM.docx]

**The guide was revised between waves based on interviewer feedback and study needs.**

**Wave 1**

I want to thank you for agreeing to participate in this study. My name is [........] and I'll be conducting this interview.

This research aims to understand the experiences of parents raising kids recently diagnosed with autism. The goal is to uncover ways the system can better support families and increase the quality of life for children and families.

Apart from this interview, we will conduct two other interviews with you later.

Please be assured that all information you share will be kept confidential. There are no significant anticipated risks associated with participating in this study, but there may be some emotional moments as we discuss sensitive topics. Please feel free to pause at any point if needed. We have resources available to provide you with additional support if you ever require it.

If you agree to participate, I would appreciate it if you could sign this consent form. Once we have completed that step, we can begin our conversation.

First, I want to ask some questions about your and your child's age, marital status, etc. and then ask some other questions.

| RESEARCH FOCUS | QUESTIONS | PROBE |
| --- | --- | --- |
| Getting the ASD Diagnosis | 1. When did you first have concerns about your child's development? | What specific behaviors or milestones first caused you to be concerned? Can you give examples? |
|  |  | How old was your child when you started noticing these issues? |
|  |  | Did you share these early concerns with one? What was their reaction or advice? |
|  | 1. How did you feel when you received the diagnosis? | What specific emotions were you experiencing when you first heard the autism diagnosis? Shock, sadness, anger? |
|  |  | Did the diagnosis confirm suspicions you already had or was it completely unexpected news? |
|  |  | How supported did you feel by the medical professionals giving the diagnosis? Did you have any unanswered questions? |
|  | 1. What challenges did you encounter during the diagnosis process? | How did you navigate the costs associated with treatment? |
|  |  | Were you able to find a suitable doctor and hospital for the diagnosis? If not, what challenges did you face? |
|  |  | What factors influenced your choice of doctor and hospital for the diagnosis? |
| Accessing post-diagnosis services | 1. What were the first steps you took after receiving the diagnosis in terms of accessing services and support? | What types of therapies or services have you considered or tried accessing for your child so far? |
|  |  | Applied for any formal autism services yet? Which ones? |
|  |  | Looked into any private therapy options? Which types? |
|  | 1. Did you do your own research to learn more about autism? What resources or information were most helpful | Could you share some details about the resources or information that you found most helpful or informative? |
|  |  | Where did you go to find out about this and what resources were available? |
|  |  | Can you give a specific example of a therapy you wanted but could not afford? About how much would it have cost? |
|  | 1. Have there been financial barriers, insurance issues, waitlists, or other obstacles? | What specific limitations or exclusions do? |
|  |  | Tell me about finances. |
|  |  | Do you have insurance? Does it cover your cost? |
|  |  |  |
|  | 1. Tell me about your child's experience in education | Is your child of school age?  Have you enrolled your child in a school or educational program? If so, can you share where? |
|  |  | What are your thoughts about finding the right educational environment for your child in both the near future and the long term? |
|  |  | What hopes or expectations do you have related to your child's future education and development needs? Do you have any concerns? |
| Barriers | 1. What would you say are some of the main barriers or challenges that you face in getting medical care and services for your child? | Can you tell me more about one of those key barriers? What specifically makes addressing that difficult? |
|  |  | How often does that particular barrier you mentioned come up? Can you give a recent example? |
|  | 1. When you encounter those types of barriers, how do you tend to deal with or overcome them? | Do you ask others for help or advice? If so, who do you reach out to? |
|  |  | Have you found any effective strategies or workarounds when facing those barriers? |
|  | 1. If you had the ability to remove or address some of those key barriers you face, which ones would have the most impact in helping you and your family? | Why is that particular barrier the most significant one to address in your view? What makes it especially challenging or impactful? |
|  |  | What difference would it make in your day-to-day life if these barriers no longer existed? Would things be much easier without facing that obstacle? Can you give examples? |
| Life changes | 1. Describe a typical day in your child's life. | Can you tell me more about their daily activities and interactions? |
|  |  | How does your child interact with family members and others in the community? |
|  | 1. What strategies have you found helpful in managing the challenges associated with your child's condition? | Do you think parent advocacy groups could help drive positive change for families affected by autism? |
|  |  | What local non-profit organizations, advocacy groups, or autism associations are you aware of or involved with? Have they been helpful? In what ways? |
|  | 1. What gives you hope when envisioning the future for your child and family? | What specific developmental, social, or educational milestones or successes are you hoping your child will achieve in the next few years? |
|  |  | Looking further ahead, how do you envision life for your child and family in the future? Do you have any worries, hopes, or expectations about how your child’s needs and this situation will evolve over time? |
|  | 1. How has caring for a child with autism impacted your relationships with your partner or other family members | Can you share an instance where caregiving led to conflict or tension? |
|  |  | In what ways has this experience brought you and your family closer together or strengthened your family bonds? |
|  |  | Tell me about the Impact of Autism on Siblings. |
| solutions | 1. At this early point after your child's diagnosis, what do you think could be helpful solutions to better support families like yours? | What types of resources or support might families need most in the immediate aftermath of a diagnosis? |
|  |  | What advice would you give families who are just starting this journey? |
|  |  |  |
|  |  |  |
|  |  |  |
|  |  |  |

Conclusion:

**How do you feel about our conversation and this interview?**

Is there anything else you feel is important for us to know that we haven't covered yet?

Wave 2

Hello, and thank you for your continued involvement in this important study. My name is ____, and I'm thrilled to have the opportunity to speak with you today.

As a reminder, the goal of this research is to identify challenges and ways to better support families and enhance the quality of life for both parents and children affected by autism.

We're conducting this interview approximately one year after after last interview to gather your insights and reflections on the first year following the diagnosis.

Please be assured that all information you share will be kept confidential. There are no significant anticipated risks associated with participating in this study, but there may be some emotional moments as we discuss sensitive topics. Please feel free to pause at any point if needed. We have resources available to provide you with additional support if you ever require it.

If you agree to participate, I would appreciate it if you could sign this consent form. Once we have completed that step, we can begin our conversation.

To start, let's briefly review any significant changes or updates that have occurred in your family over the past year. Then, we can transition into discussing the key aspects of your journey that I'd like to focus on in this interview."

| RESEARCH FOCUS | QUESTIONS | PROBE |  |
| --- | --- | --- | --- |
| School and Social Interactions | 1. How has your child's experience in educational settings changed since our last discussion? | In what areas are they progressing steadily in terms of expected social development? Are there areas where their development falls short of your hopes? |  |
|  |  | Why do you believe some aspects of their social skills are developing more rapidly or slowly than anticipated? |  |
|  |  | Are there any new developments in your child's social interactions that you would like to share? |  |
|  | 1. How have your child's social interactions at school evolved over the past year? | Has their capacity or willingness to engage with peers improved, declined, or remained consistent? |  |
|  |  | Are they participating more, less, or the same in group activities compared to a year ago? |  |
|  |  | What new challenges or improvements have teachers reported regarding your child's social skills? |  |
|  | 1. Beyond the school setting, what types of social interactions does your child engage in within the community and neighborhood? | Approximately how often does your child interact or play with other children outside of school? Has this frequency increased or decreased over the past year? |  |
|  |  | What obstacles, if any, continue to hinder the development of friendships or playdates for your child? |  |
| Access to Services and support | 1. Can you discuss any experiences, positive or negative, with healthcare providers and support services since our last conversation? | Can you elaborate on any specific instances where you felt that healthcare providers or support services were particularly helpful or supportive? |  |
|  |  | **Have you encountered any new obstacles or hindrances in accessing services for your child in the past year?** |  |
|  |  | **What progress have you observed over the past year of therapy for your child? Are there any new skills or improvements that you've noticed?** |  |
|  |  | What therapies is your child currently receiving? |  |
| Stigma and awareness | 1. What kinds of unpleasant statement or misconceptions about autism have you encountered from others? | What are the most common stereotypes you’ve heard? |  |
|  |  | How do those stereotypes make you feel? |  |
|  |  | Have you experienced any discrimination due to your child’s autism diagnosis? |  |
|  | 1. In what ways do you think greater public awareness and understanding of autism could help combat some of that stigma? | What key facts or messages would be most important for the public to understand? |  |
|  |  | How could increased awareness translate to reduced discrimination? |  |
|  | 1. Do you ever find yourself needing to educate teachers, relatives, friends or strangers to correct misconceptions about autism or your child? | What types of misconceptions have you needed to correct? |  |
|  |  | How do you approach educating someone when the need arises? |  |
|  |  | In what ways could better public awareness help reduce that burden on parents? |  |
| life changes | 1. How has caring for a child with autism affected your relationships with your partner or other family members? | In what ways has having a child with autism further impacted or changed family life over the past year? |  |
|  |  | What new challenges related to your child has the family faced in areas like finances, marital relationship, sibling needs, your own career or personal goals? |  |
|  | 1. How are you mentally and physically? | Can you describe your current state of mind and overall mental well-being? |  |
|  |  | Have there been any notable changes or concerns regarding your physical well-being recently? |  |
|  | 1. What strategies have proven effective in handling the challenges associated with your child's condition? | Do you maintain the belief that parenting support groups can contribute to positive changes for families dealing with autism? Have there been any notable developments in this area? |  |
|  |  | Regarding local autism nonprofits, advocacy groups, or associations, have you observed any changes since last year? If so, have they been beneficial, and in what ways have they provided support? |  |
| Reflection | 1. You were initially worried about ____. How are things going related to that original worry? Any improvement? |  |  |
|  | 1. When we first interviewed you, you hoped _____. Do you feel that hope has been realized over the past year? Why or why not? |  |  |

Conclusion:

1. How do you feel about what we discussed in this interview?
2. Do you have anything further to add?

Wave 3

Hello, and thank you for your continued involvement in this important study. My name is ____, and I'm thrilled to have the opportunity to speak with you today.

As a reminder, the goal of this research is to identify challenges and ways to better support families and enhance the quality of life for both parents and children affected by autism.

We're conducting this interview approximately two years after your child's initial diagnosis to gather your insights and reflections on the two years following the diagnosis.

Please be assured that all information you share will be kept confidential. There are no significant anticipated risks associated with participating in this study, but there may be some emotional moments as we discuss sensitive topics. Please feel free to pause at any point if needed. We have resources available to provide you with additional support if you ever require it.

You have now been through over 2 years of raising a child diagnosed with autism. We started this process shortly after your child’s initial diagnosis to capture those early challenges and emotions. Now at the 30 month mark post-diagnosis, I’d love to discuss your perspectives looking back at the full trajectory since then. Please interject at any point if you need to take a break or stop the interview. Everything shared will remain fully confidential.

For this final wave, I want to devote time to reflecting on the entire journey - how diagnosis and subsequent experiences have impacted your child and family over time. We’ll recap key parts of your story, discuss what stands out looking back and talk about how your outlook and priorities may have shifted, and also look ahead to the future. We begin by briefly recapping anything major that has changed recently and then delve more deeply into your recollections of the past couple of years. Please feel free to share and reflect in any way you feel comfortable.

If you agree to participate, I would appreciate it if you could sign this consent form. Once we have completed that step, we can begin our conversation.

| RESEARCH FOCUS | QUESTIONS | PROBE |  |
| --- | --- | --- | --- |
| School and Social Interactions | 1. How has your child's experience in educational settings changed since our last discussion? | In what areas are they progressing steadily in terms of expected social development? Are there areas where their development falls short of your hopes? |  |
|  |  | Why do you believe some aspects of their social skills are developing more rapidly or slowly than anticipated? |  |
|  |  | Are there any new developments in your child's social interactions that you would like to share? |  |
|  | 1. How have your child's social interactions at school evolved over the past year? | Has their capacity or willingness to engage with peers improved, declined, or remained consistent? |  |
|  |  | Are they participating more, less, or the same in group activities compared to a year ago? |  |
|  |  | What new challenges or improvements have teachers reported regarding your child's social skills? |  |
|  | 1. Beyond the school setting, what types of social interactions does your child engage in within the community and neighborhood? | Approximately how often does your child interact or play with other children outside of school? Has this frequency increased or decreased over the past year? |  |
|  |  | What obstacles, if any, continue to hinder the development of friendships or playdates for your child? |  |
| Access to Services and support | 1. Can you discuss any experiences, positive or negative, with healthcare providers and support services since our last conversation? | Can you elaborate on any specific instances where you felt that healthcare providers or support services were particularly helpful or supportive? |  |
|  |  | **Have you encountered any new obstacles or hindrances in accessing services for your child in the past year?** |  |
|  |  | **What progress have you observed over the past year of therapy for your child? Are there any new skills or improvements that you've noticed?** |  |
|  |  | What therapies is your child currently receiving? |  |
| Stigma and awareness | 1. What kinds of unpleasant statement or misconceptions about autism have you encountered from others? | What are the most common stereotypes you’ve heard? |  |
|  |  | How do those stereotypes make you feel? |  |
|  |  | Have you experienced any discrimination due to your child’s autism diagnosis? |  |
|  | 1. In what ways do you think greater public awareness and understanding of autism could help combat some of that stigma? | What key facts or messages would be most important for the public to understand? |  |
|  |  | How could increased awareness translate to reduced discrimination? |  |
|  | 1. Do you ever find yourself needing to educate teachers, relatives, friends or strangers to correct misconceptions about autism or your child? | What types of misconceptions have you needed to correct? |  |
|  |  | How do you approach educating someone when the need arises? |  |
|  |  | In what ways could better public awareness help reduce that burden on parents? |  |
| life changes  Regional inequalities | 1. How has caring for a child with autism affected your relationships with your partner or other family members? | In what ways has having a child with autism further impacted or changed family life over the past year? |  |
|  |  | What new challenges related to your child has the family faced in areas like finances, marital relationship, sibling needs, your own career or personal goals? |  |
|  | 1. How are you mentally and physically? | Can you describe your current state of mind and overall mental well-being? |  |
|  |  | Have there been any notable changes or concerns regarding your physical well-being recently? |  |
|  | In our previous interview, you mentioned regional inequalities. Could you provide more details about this topic? Specifically, how has the situation changed over the past year? | Can you elaborate on any specific challenges or improvements you've observed regarding regional access to healthcare and support services for autistic children since our last conversation? |  |
|  |  | How have community perceptions and awareness about autism evolved in your region, and how do you think these changes have influenced the experiences of families dealing with autism? |  |
| Reflection | 1. You were initially worried about ____. How are things going related to that original worry? Any improvement? |  |  |
|  | 1. When we first interviewed you, you hoped _____. Do you feel that hope has been realized over the past year? Why or why not? |  |  |

Conclusion:

1. How do you feel about what we discussed in this interview?
2. Do you have anything further to add?
